# Radiological Findings of COVID-19 Patients in Italy

**DOI:** 10.51894/001c.14505

**Published:** 2020-10-30

**Authors:** Zachary Brennan, Samantha Guerra, Susan Seman

**Affiliations:** 1 Michigan State University College of Osteopathic Medicine; 2 0000-0003-3443-7422 Michigan State University College of Osteopathic Medicine; 3 Detroit Medical Center - Sinai Grace Hospital

**Keywords:** computed tomography, covid-19, sars-cov2, radiology, radiograph, x-ray, diagnosis

## Abstract

**CONTEXT:**

The emergence of COVID-19/SARS-CoV2 (COVID-19) was an outbreak that began in December 2019 and rose to pandemic levels in 2020. One of the largest problems with COVID-19 is the typical delay in testing and diagnosis that can lead to additional transmission of the disease. Under consultation with a board-certified radiologist, the study team evaluated the common radiological findings of COVID-19 on computed tomography (CT) and compared the efficacy of chest radiographs (i.e., x-rays) to CT in diagnosing COVID-19.

**METHODS:**

In 2020, the authors completed a retrospective review of radiologic imaging data (i.e., the original imaging report notes) from Italy performed on 47 patients who had tested positive for COVID-19 in Italy during the national outbreak from February to March 2020. Radiologic images were obtained from Società Italiana di Radiologia Medica e Interventistica radiological database of COVID-19 patients. Each case was analyzed for whether they had positive findings on either chest radiograph or CT or both among patients who had positive COVID-19 test results.

**RESULTS:**

The authors found significant radiological finding similarities among the 47 COVID-19 positive case studies from Italy during the February to March 2020 time period. Ground glass opacities and crazy paving were the most significant findings, resembling the findings in China and other Coronavirus strains. The authors’ statistical analyses indicated that CT scans were more reliable by 30.7% than chest radiographs in identifying signs of COVID-19. In cases where either an initial negative swab for COVID-19 or providers lacked patient social histories, chest radiographs were used to show clinical findings consistent with COVID-19.

**CONCLUSIONS:**

Based on these results, chest radiographs appear to be a consistent method to assist in the diagnosis of most COVID-19 cases. The authors discuss several scenarios in community-based and non-hospital US settings for COVID-19 diagnostic processes.

## INTRODUCTION

During 2020, the COVID-19/SARS-CoV2 (COVID-19), sometimes referred to as the “coronavirus,” has been the cause of a major viral respiratory infection global pandemic.^1^ At the time of this publication, the US Centers for Disease Control and Prevention (CDC) had listed the following symptoms associated with COVID-19 infections: fever, chills, cough, shortness of breath, fatigue, body aches, headache, new onset of loss of taste or smell, sore throat, congestion, runny nose, nausea/vomiting, and diarrhea.^1^

When testing resources have been limited, patients with mild symptoms and no co-morbidities in some settings have been sent home without COVID-19 testing since the virus may resolve on its own.^2,3^ Those patients who are sent home should be advised to self-quarantine for 14 days to prevent spreading to others.^2^ Patient who have cardiovascular disease, chronic lung disease, hypertension, diabetes, obesity, or over the age of 65 should generally be further evaluated.^2^ Patients who experience a sudden decline, especially in lung function, should also be admitted to the hospital for further evaluation.^2,3^

Patients with moderate or severe symptoms should generally be hospitalized. Moderate COVID-19 infection has been defined as having dyspnea (i.e., shortness of breath) but having a blood oxygen saturation level of 94%.^3^ Severe COVID-19 infection have been defined as someone with marked tachypnea (i.e., increased respirations), hypoxemia (i.e., inadequate oxygenation), and lung infiltrates.^3^ Unfortunately, there has yet to be any validated treatments for COVID-19 at the time of this publication.

Patients who are hospitalized generally need to be frequently monitored and provided supportive respiratory therapy (i.e., non-invasive ventilation or continuous positive airway pressure when possible, followed by invasive ventilator if the patient worsens).^4,5^ Until there is an approved COVID-19 treatment, individuals are advised to continue to self-isolate, wash their hands frequently, and wear the appropriate personal protective equipment (PPE) when around those infected with COVID-19.^3^

To date, the virus has imposed a significant strain on hospital systems, showing a profound impact on daily life in affected areas and death rate higher than influenza.^6^ One of the ongoing difficulties of containing this virus in some parts of the US has been the lack of reliable methods and testing supplies to reliably diagnose the virus.^7^

There are several types of swab testing for COVID available. The main types are oropharyngeal and nasopharyngeal swabs with a reverse transcriptase polymerase chain reaction (RT-PCR) test on the swab results.^8,9^ The nasal or nasopharyngeal swab is inserted farther into the nostril on an imaginary line pointed to the ear; the oropharyngeal or oral swab is less invasive and involves touching the back of the throat with the swab. Recent studies have shown how important it is to properly collect the sample in order to avoid a false negative.^8,10^

To date, there have been several studies demonstrating common findings concerning COVID-19 on chest computed tomography (CT) scans.^11^ Two recent Chinese studies have explored whether CT was a reliable method in diagnosing COVID-19, determining that it was a useful diagnostic method.^12,13^ During these studies, CT was also successful in diagnosing when RT-PCR genetic testing showed a negative finding but clinical symptoms were consistent with COVID-19.^12,13^ In Hubei, China, during the COVID-19 outbreak in early 2020, CT was temporarily adopted as the standard for diagnosing COVID-19 due to the procedure’s higher relative sensitivity than chest radiographs (i.e., x-rays).^14^

Since early 2020, the Società Italiana di Radiologia Medica e Interventistica has been cataloging radiological images during the COVID-19 outbreak in Italy. For the study described in this paper, the authors investigated the common findings among these pre-existing radiology reports. They compared differences in CT and x-ray imaging modality results and evaluated the use of chest radiographs to show positive COVID-19 findings when other methods were not available.

## METHODS

Under consultation with a board-certified radiologist, the authors analyzed a total of N = 47 case reports that had been previously published on the Società Italiana di Radiologia Medica e Interventistica COVID-19 database. These case reports were analyzed for data regarding commonalities between radiological findings of patients who later tested positive for COVID-19. One case report was excluded because the patient had not been swabbed for COVID-19 and was sent home after they received a CT with minimal findings. Two additional case reports were excluded because they had not contained the radiologists’ notes with the CT report.

During this study, the three main types of imaging abnormalities include: a) ground glass opacities (i.e., hazy opacities that don’t obscure the underlying structures such as vessels), b) interlobular septa thickening (i.e., appearance of lines between lobes of the lung being more pronounced than usual),^15^ and c) crazy paving (i.e., finding of ground glass opacities with superimposed interlobular septa or intralobular septa thickening).^16^

The authors assessed each case based on the findings of the chest CT, chest radiograph, or both. They also tabulated whether the radiologist noted findings such as ground glass opacities, crazy paving, or other results. Finally, they assessed when it was noted by radiologists that chest radiographs had demonstrated absent or inconclusive results but CTs had provided positive findings for a COVID-19 diagnosis.

All data were based upon the Italian radiologists’ imaging original report notes (i.e., not the authors’ interpretation of the radiological images). Statistical analyses were completed using a two-sample proportion test of the analytic software program *R*^17^ with the assistance of Michigan State University Center for Statistical Training and Consulting.

## RESULTS

**Figure 1.**
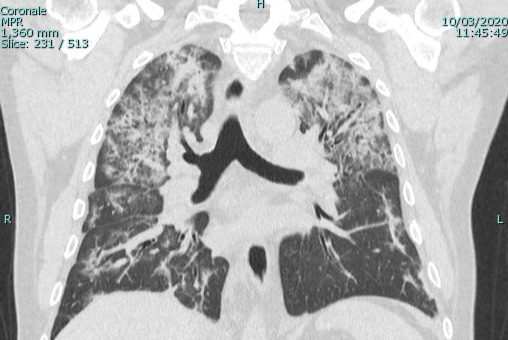
Ground Glass Opacities

**Figure 2.**
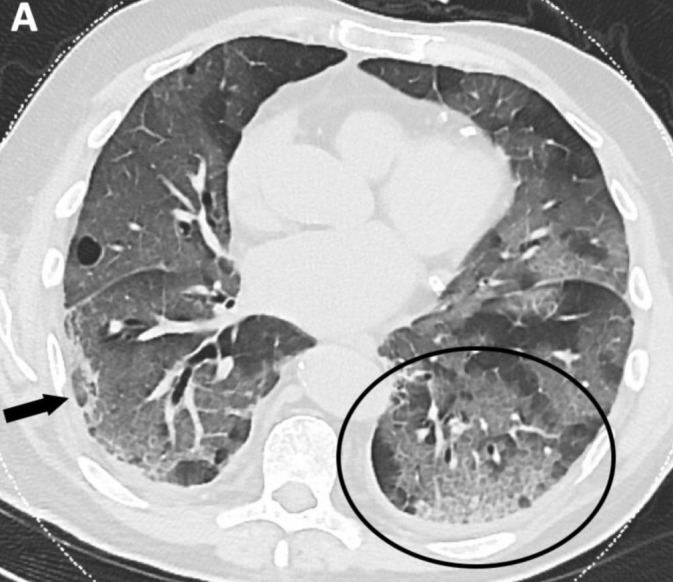
Ground Glass Opacities with Crazy Paving

Of the total 47 cases, there were 16 (34.0%) case reports with only a CT, two (4.2%) with only a chest radiograph, and 26 (55.3%) with both a CT and a chest x-ray. Ground glass opacities were noted for 33 (69.8%) of patients with a CT (Figure 1). Among case reports with a CT published, crazy paving was noted by the radiologist in 10 (20.9%) of the case reports (Figure 2) and thickening of the interlobular septa was noted by the radiologist in 14 (30.2%) of the case reports.

**Figure 3.**
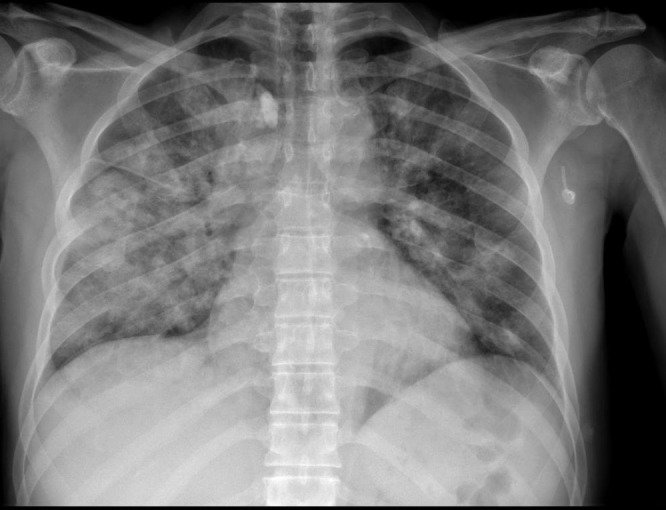
Radiograph the radiologist stated showed positive findings for COVID-19

**Figure 4.**
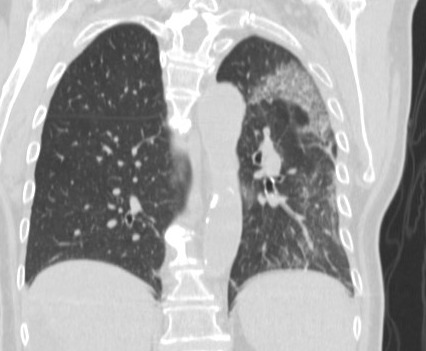
COVID-19 positive patient with findings in left lung only

In the 33 (69.3%) of the cases with both a chest CT and a chest radiograph, the radiograph showed signs of COVID-19 (Figure 3). Most chest radiographs had non-specific findings, with the most common findings noted as hypotransparency (i.e., mild opacity in an area that should be transparent) or the radiologist documenting that the chest x-ray was consistent with COVID-19 without further detail. A total of 46 (97.8%) of the sample cases showed bilateral lung findings consistent with COVID-19 on CT. One (2.1%) patient x-ray showed only unilateral findings, with ground glass opacities being found in the left lung only. (Figure 4)

**Figure 5.**
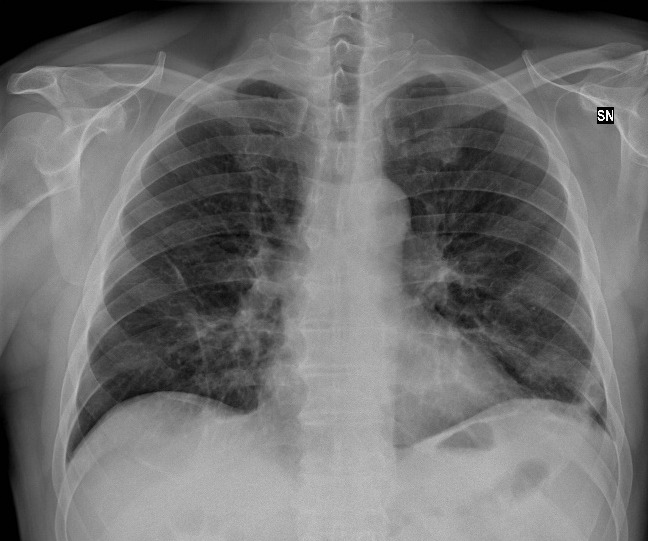
Radiologist declared radiograph negative (same patient as Figure 6)

**Figure 6.**
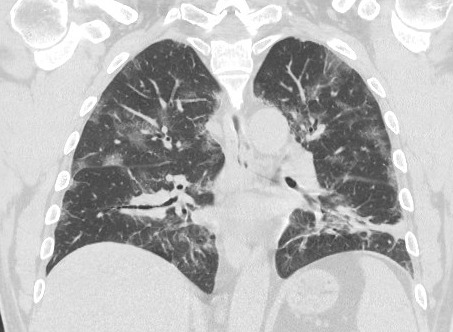
Radiologist notes findings consistent with COVID-19 (same patient as Figure 5)

One (2.1%) sample patient tested negative via nasal swab for COVID-19, but continued symptoms led to a physician ordering a chest CT, which showed bilateral ground glass opacities; this led to a second swab, which was positive for COVID-19. Both figures 5 and 6 are from this patient, showing a chest radiograph that the radiologist deemed negative and a chest CT that was deemed by a radiologist to have positive findings which is what led to a second nasal swab. (Figures 5 and 6)

In another somewhat similar sample case, a patient was not swabbed because of a lack of social history demonstrating exposure to the virus. A CT was later ordered due to the continued symptoms in the patient and this additional imaging showed bilateral ground glass opacities, which led to a swab test. The swab test resulted in a positive finding for COVID-19.

Among the cases where both a chest radiograph and a chest CT were done, 14 (30.7%) of these cases resulted in negative or inconclusive findings on chest radiograph. In these cases, a chest CT was ordered as follow up based on clinical symptoms, demonstrating significant radiological findings consistent with COVID-19. CT showed signs of COVID-19 in 100% of sample patients receiving both a chest CT and a chest x-ray.

Each of these patients all tested positive for COVID-19 on a later nasal swab. This indicates a 95% CI of .50 to .83 (p < 0.001) for chest radiograph in identifying COVID-19 infection, and a 95% CI of .87 and 1 (p < 0.001) for CT in identifying COVID-19 infection.

## DISCUSSION

According to an earlier-cited 2020 Chinese study, the common findings among COVID-19/SARS-CoV-2 patients were similar to other coronavirus strains.^11^ However, it has since been concluded that COVID-19 seems to result in a more severe pneumonia than most earlier common strains of coronavirus like SARS-CoV-1 and MERS-CoV.^15^

In our sample cases, ground glass opacities were found in 33 (69.8%) patients, crazy paving was found in 10 (20.9%) of cases and thickening of the interlobular septa was found in 14 (30.2%) of all cases. These proportions are each consistent with a 2020 radiological study focusing on CT with 81 patients in China.^18^ Although our study findings aren’t specifically novel, they confirm that CT can be used to establish an initial COVID-19 diagnosis, especially when other diagnostic modalities have failed to confirm the condition and/or patients’ clinical presentations may be ambiguous.

Based on another early 2020 Chinese study conducted during the initial outbreak, RT-PCR genetic testing did not seem to be totally effective at initial onset of symptoms, with between a 43% and 61% successful diagnosis rate.^14^ In a slightly later study, chest radiographs were shown to be useful in showing radiological findings consistent with COVID-19, but were less reliable than CT.^19^

Similar studies have also shown a high degree of sensitivity for diagnosing viral pneumonia as well as similar levels of ground glass opacities, especially early in the course of the disease.^20–22^ One 2020 study showed similar results to our study for both the radiograph and CT results, though this group additionally noted enlarged lymph nodes in 14.3% of patients and pleural effusion in 7.1% of patients.^23^

It is important to consider that there is typically less access to CT testing in rural America, with many COVID-19 patients often at higher risk because they are more often older and have a higher rates of co-morbidities.^24^ When determining how to treat suspected COVID-19 patients under such resource constraints, it has remained important to gauge how severe their symptoms are.^24^

Realistically, it can be challenging for many clinicians to obtain thorough social histories from patients who had been in contact with known COVID-19 positive persons. Contact tracing is such a method used to track the spread of infection from individual to individual to implement quarantine as an effective method to stop the spread of the virus.^25^ As soon as a patient starts to show possible COVID-19 symptoms, they should be tested using the nasopharyngeal swab test and RT-PCR testing.^26^

If a nasopharyngeal swab test comes back negative but the patient continues to show symptoms associated with COVID-19, they should be swabbed again due to the still unknown rate of possible false negatives associated with the nasopharyngeal swab test.^3,26^ The CDC has also indicated that when a nasopharyngeal swab test cannot be administered (most likely due to lack of supplies), an oropharyngeal swab can be used in its place.^3,26^

There has also been some empiric indications that there may be two distinct strains of this virus, especially when comparing Chinese positives with Italian positives.^27^ If this is confirmed to be the case, the findings from this study would likely only apply to the strain that was prevalent in Italy, as other findings and correlations might not be true for the other strain. More research examining different COVID-19 strains and their radiological findings will be necessary to determine whether or how these two strains might distinctly present clinically and/or radiologically.^28^

### Study Limitations

There were several incumbent limitations associated with our study. This study had a small sample size using secondary Italian radiologic interpretation data. The interpretation of our study results may also be limited since these 47 COVID-19 cases may not have been randomly drawn from the entire population of afflicted patients in Italy.

## CONCLUSIONS

Further research into the relative diagnostic utility of CT and radiograph in diagnosing COVID-19 in varied clinical care settings is required to attain an improved understanding of how to control COVID-19 outbreaks. Based on these study results, chest x-rays should remain an important initial diagnostic modality for COVID-19 in several ways. Chest radiographs may help augment other methods of diagnosis (e.g., nasopharyngeal swab testing, chest CT) as a follow-up when other methods of diagnosis are not readily available, as an aid in determining the severity of the disease.

Because chest x-rays are cheaper and often more accessible, they may be warranted when a suspected COVID-19 patient presents in an office or emergency room setting.^27^ Although a negative or inconclusive chest radiograph should not be interpreted as absolutely negative for a COVID-19 diagnosis, a follow up CT could be later ordered if a patients’ symptoms worsen. Further research in this area is certainly required to better understand the optimal roles of radiological imaging options during our nation’s complex ongoing COVID-19 outbreaks.

**Acknowledgements**: Nicole Jess, Research Assistant, PhD(c), Michigan State University Center for Statistical Training and Consulting for her assistance with statistical analysis.

Michigan State University Department of Radiology, Colleges of Osteopathic Medicine, Division of Human Anatomy for their consultative assistance with study design and analysis.

The authors report no external funding source for this study.

The authors declare no conflict of interest.

Submitted for publication May 2020.

Accepted for publication August 2020.
